# Effect of High-Energy Ball Milling, Capping Agents and Alkalizer on Capacitance of Nanostructured FeOOH Anodes

**DOI:** 10.3390/nano13101693

**Published:** 2023-05-21

**Authors:** Chengwei Zhang, Igor Zhitomirsky

**Affiliations:** Department of Materials Science and Engineering, McMaster University, Hamilton, ON L8S 4L7, Canada; zhanc14@mcmaster.ca

**Keywords:** supercapacitor, anode, iron hydroxide, capping agent, alkalizer

## Abstract

This investigation is motivated by interest in nanostructured FeOOH anodes for aqueous asymmetric supercapacitors operating in Na_2_SO_4_ electrolyte. The research goal is the fabrication of anodes with high active mass loading of 40 mg cm^−2^, high capacitance and low resistance. The influence of high-energy ball milling (HEBM), capping agents and alkalizer on the nanostructure and capacitive properties is investigated. HEBM promotes the crystallization of FeOOH, which results in capacitance reduction. Capping agents from the catechol family, such as tetrahydroxy-1,4-benzoquinone (THB) and gallocyanine (GC), facilitate the fabrication of FeOOH nanoparticles, eliminate the formation of micron size particles and allow the fabrication of anodes with enhanced capacitance. The analysis of testing results provided insight into the influence of the chemical structure of the capping agents on nanoparticle synthesis and dispersion. The feasibility of a conceptually new strategy for the synthesis of FeOOH nanoparticles is demonstrated, which is based on the use of polyethylenimine as an organic alkalizer-dispersant. The capacitances of materials prepared using different nanotechnology strategies are compared. The highest capacitance of 6.54 F cm^−2^ is obtained using GC as a capping agent. The obtained electrodes are promising for applications as anodes for asymmetric supercapacitors.

## 1. Introduction

Various electrode materials are currently under investigation for application in supercapacitors [1,2,3,4]. FeOOH is a promising energy storage material for anodes of asymmetric aqueous supercapacitors [5]. The capacitive properties of this material in the negative potential range are related to Fe^3+^/Fe^2+^ redox reactions [6]. The capacitive performance of different phases was analyzed, including α-FeOOH [7], β-FeOOH [5,8], γ- FeOOH [9] and an amorphous phase [10]. It was found that capacitive properties are influenced by phase content [7,10]. Of particular importance are particle size and morphology. The use of nanoparticles with a high surface area facilitated electrolyte access to the particle surface and resulted in high capacitance [6]. Therefore, significant efforts have focused on the fabrication of non-agglomerated FeOOH nanoparticles [7]. It was found that the reduction iin nanoparticle size resulted in enhanced capacitance [6]. Many investigations have targeted the fabrication of doped FeOOH with the goal of improving electrical conductivity and cyclic stability [8,11]. Electrochemical testing was performed in different electrolytes, such as Li_2_SO_4_ [5], ionic liquid [9], KOH, Na_2_SO_3_ and Na_2_SO_4_ [6]. KOH and LiOH electrolytes offer the advantages of high solubility and high conductivity, which are important for energy storage in supercapacitors [12,13,14]. However, Na_2_SO_4_ is a more environmentally friendly electrolyte, which offers benefits for the fabrication of asymmetric devices with an enlarged voltage window of 1.6–2.0 V [6,7,11,15].

Enhanced electrochemical performance of FeOOH-based anodes was obtained by the fabrication of nanocomposite materials. The problem of poor electrical conductivity of FeOOH [6] was addressed by the use of nanocomposites containing carbon materials, such as carbon nanotubes, graphene and carbon black [6,7]. Good electrochemical performance was achieved for FeOOH nanocomposites containing polyaniline [16], polypyrrole [17], metal oxides and layered double metal hydroxides [18,19]. Recent studies [20] emphasized the need for electrodes with high active mass loading, a high ratio of active material mass to the mass of the current collector, high areal capacitance (C_S_, Fcm^−2^) and low resistance, which are critically important for practical applications. However, due to the diffusion limitation of the electrolyte, the specific capacitance (C_m_, Fg^−1^) of electrode materials decreases with increasing active mass, resulting in a low C_S_. The important challenge is to obtain high C_S_ and low electrode resistance. This can be achieved by the development of advanced nanotechnologies for the fabrication of nanoparticles and their efficient mixing with conductive additives on the nanometric scale.

Wet chemical synthesis methods represent an important tool in nanotechnology. Such methods are widely used in the nanotechnology of metals, quantum dots, metal oxides and hydroxides for nanoparticle synthesis from solutions of metal salts [21]. Such methods offer the advantages of simplicity, affordability, speed and good control of particle size and morphology. The enhanced performance characteristics of nanomaterials prepared by wet chemical methods afford significant advantages for supercapacitor applications [22,23,24,25]. However, particles prepared by wet chemical methods are prone to agglomeration and particle growth due to Ostwald ripening. Therefore, in this investigation, we developed different strategies for the fabrication of nanoparticles with reduced particle size and low agglomeration. This investigation is motivated by increasing interest in high-energy ball milling for the reduction of particle size. Another promising strategy is based on the use of new capping agents for synthesis, which can limit particle growth and prevent agglomeration. The feasibility of avoiding the use of inorganic alkalis for wet chemical synthesis by the use of organic alkalizers-dispersants is another promising strategy, which was explored in this investigation.

The goal of this investigation was to fabricate and test advanced FeOOH-carbon nanotube anodes for supercapacitors. The strategies explored in this investigation included high-energy ball milling, application of advanced capping agents and the use of organic alkalizers-dispersants. We described the fundamental mechanisms and compared the performance of electrodes prepared using different methods. The experimental results presented below show the enhanced performance of electrodes, which are promising anodes for asymmetric supercapacitors.

## 2. Materials and Methods

FeCl_3_·6H_2_O, NaOH, Na_2_SO_4_, poly(vinyl butyral) (PVB, M_W_ = 150,000), polyethylenimine (PEI, M_W_ = 110,000), tetrahydroxy-1,4-benzoquinone (THB), gallocyanine (GC) (MilliporeSigma, Oakville, ON, Canada), multiwalled carbon nanotubes (MWCNTs, OD 13 nm, ID 4 nm, length 1–2 μm, Bayer, Leverkusen, Germany) and Ni foams (thickness 1.6 mm, 95% porosity, Vale, Mississauga, ON, Canada), were used. Stock solutions (SS) were prepared by dissolving 10 g L^−1^ FeCl_3_·6H_2_O in DI water.

FeOOH active material (FAM) was prepared from SS by pH adjustment to pH = 7 using aqueous 2M NaOH. The obtained precipitate was washed with DI water and dried.

FAM-BM was prepared using the same precipitation procedure and high-energy ball milling of the precipitate. High-energy ball milling (HEBM) was performed using a Mixer Mill MM 500 Nano (Retsch GmbH, Haan, Germany) at a frequency of 15 Hz. The milling procedure consisted of milling steps with a duration of 5 min and 1.5 min interruptions between the milling steps, with a total milling duration of 2 h. After the milling process, the precipitate was washed with DI water and dried.

FAM-THB and FAM-GC were prepared from SS, containing THB and GC as caping agents, respectively. The ratio of FeCl_3_ mass to the mass of the capping agents was 100:5. The pH of the solutions was adjusted to pH = 7 by adding NaOH. The obtained precipitate was washed with DI water and dried.

FAM-PEI was prepared from SS solutions. The pH of the solutions was adjusted to pH = 7 by adding PEI as an alkalizer. The obtained precipitate was washed with DI water and dried. All materials were dried in air at 45 °C for 24 h.

The obtained active materials (AM), such as FAM, FAM-BM, FAM-THB, FAM-GC and FAM-PEI, were used for the fabrication of slurries in ethanol solvent for impregnation of Ni foam current collectors. The obtained materials showed different colloidal stabilities. FAM and FAM-BM showed significant sedimentation 2 h after ultrasonication. FAM-THB and FAM-GC were stable for more than 24 h, and FAM-PEI was stable for more than 48 h. The slurries contained MWCNT as conductive additives and PVB binder in the mass ratio of AM:MWCNT:PVB = 80:20:3. For the preparation of slurries, the PVB binder was dissolved in ethanol. The total mass of the impregnated material after drying was 40 mg cm^−2^. The impregnated foams were pressed to 38% of the original thickness to improve contact of the AM and current collector. The area of the electrodes was 1 cm^2^. Electron microscopy investigations were performed using a Talos 200 transmission electron microscope (Thermo Fisher Scientific, Waltham, MA, USA) (TEM). X-ray diffraction (XRD) analysis (diffractometer Bruker D8, Bruker, Billerica, MA, USA) was performed at a rate of 0.01 degrees per second using Co radiation.

In total, 0.5 M of Na_2_SO_4_ was used as an electrolyte. The electrodes were tested by cyclic voltammetry (CV) and electrochemical impedance spectroscopy (EIS) using a PARSTAT 2273 potentiostat (Ametek, Berwyn, PA, USA) and galvanostatic charge-discharge (GCD) using a BioLogic VMP 300 potentiostat (Biologic, Willow Hill, IL, USA). The potential range for FeOOH was −0.8 to 0 V. EIS was tested in the frequency range of 10 mHz to 100 kHz at a voltage amplitude of 5 mV. A 3-electrode electrochemical cell was used, which contains a working electrode, a reference electrode (SCE, saturated calomel electrode) and a counter-electrode (Pt mesh). The areal capacitance C_S_ (F cm^−2^) and gravimetric capacitance C_m_ (F g^−1^) were calculated from CV, EIS and GCD data, as described in a previous investigation [20].

## 3. Results and Discussion

**Figure 1:**
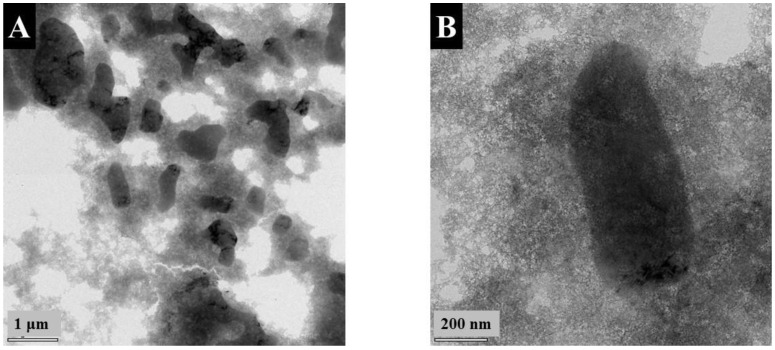
(**A**,**B**) TEM images at different magnifications: (**A**) ×8600 and (**B**) ×46,000 for as-precipitated FAM.

Figure 1 presents TEM images of FAM at different magnifications. The images show many agglomerated nanoparticles and large particles with a typical size of 0.5–2 μm. The formation of large particles can explain the poor colloidal stability of FAM suspensions. Such large particles are detrimental to the fabrication of supercapacitor electrodes. Particle size reduction is necessary for the improvement of their contact with electrolytes and improved mixing with conductive MWCNT on the nanometric scale. Such large particles can be formed by the Ostwald ripening mechanism.

**Figure 2:**
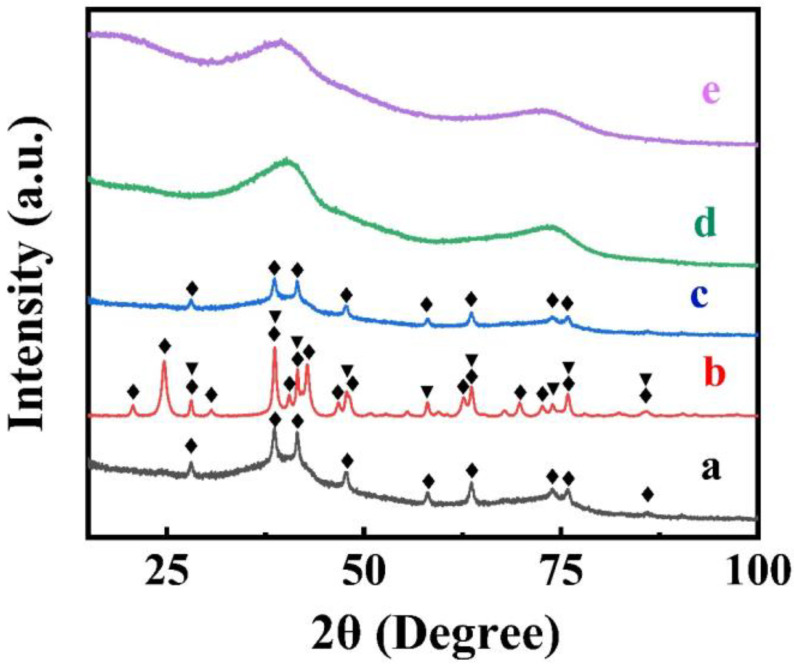
X-ray diffraction patterns of (**a**) FAM, (**b**) FAM-BM, (**c**) FAM-THB, (**d**) FAM-GC and (**e**) FAM-PEI (♦—peaks corresponding to JCPDS file 01-073-9835 of α -FeOOH, ▼—peaks, corresponding to JCPDS file 01-089-0596 of α -Fe_2_O_3_).

X-ray diffraction studies (Figure 2) of dried FAM powders showed small peaks of α-FeOOH (JCPDS file 04-015-6946). However, the FAM material also contained an amorphous phase. The XRD pattern of FAM-BM showed enhanced crystallization, as indicated by the increasing intensity of the α-FeOOH peaks and the appearance of α -Fe_2_O_3_. The enhanced crystallization and transformation to the α -Fe_2_O_3_ phase can be attributed to local heating of the material during high-energy ball milling.

**Figure 3:**
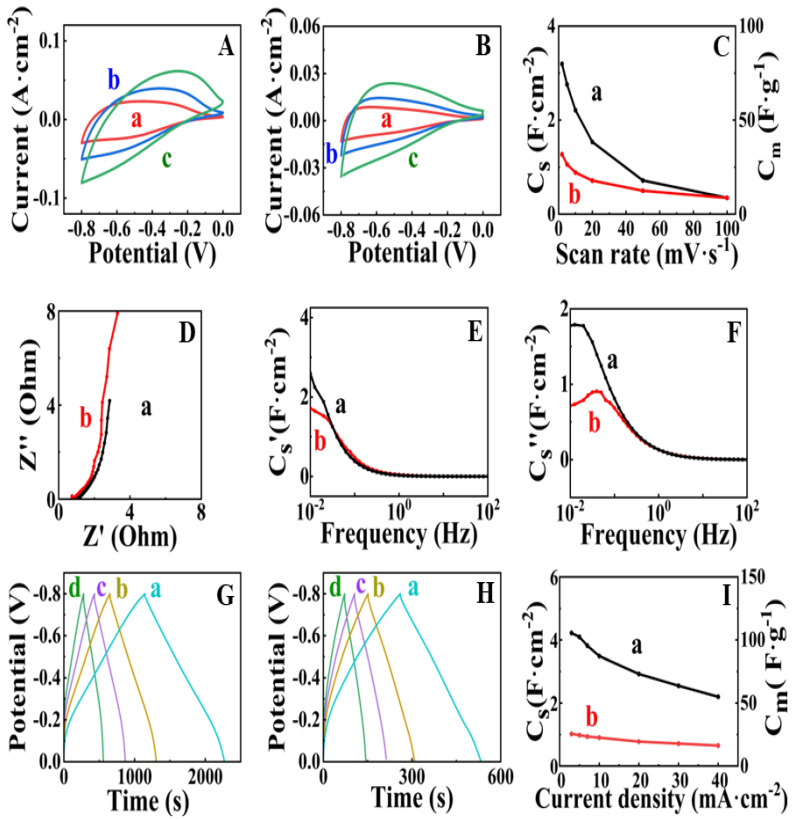
(**A**,**B**) CVs at scan rates of (a) 5, (b) 10 and (c) 20 mV·s^−1^ for (**A**) FAM and (**B**) FAM-BM and (**C**) capacitance obtained from CV data versus scan rate for (a) FAM and (b) FAM-BM; (**D**–**F**) EIS data for (a) FAM and (b) FAM-BM; (**D**) Nyquist plot of complex impedance Z*; (**E**) real C_s_′ and (**F**) imaginary C_s_″ components of complex capacitance versus frequency; (**G**,**H**) GCD data at current densities of (a) 3, (b) 5, (c) 7 and (d) 10 mA cm^−2^ for (**G**) FAM and (**H**) FAM-BM; (**I**) capacitance obtained from GCD data for (a) FAM and (b) FAM-BM. SCE was used as a reference electrode.

Figure 3 shows the electrochemical testing results for FAM and FAM-BM electrodes. Both electrodes showed poor capacitive behavior in the potential range of 0–−0.2 V, as indicated by low currents and a narrow CV shape in this range (Figure 3A,B). The FAM-BM electrodes showed smaller CV areas compared to the CV areas for FAM at the same scan rates. As a result, the FAM-BM electrodes showed lower capacitance (Figure 3C). The capacitances of FAM and FAM-BM electrodes were found to be 3.20 and 1.27 F cm^−2^, respectively, at a scan rate of 2 mV s^−1^. The capacitance decreased significantly with increasing scan rate due to diffusion limitations in the pores. A similar reduction in capacitance was observed in other investigations of cathodes and anodes [7,26]. Capacitance retention at high scan rates is a subject of significant attention for researchers involved in supercapacitor development. The EIS data (Figure 3D) showed a higher real part of impedance at a frequency of 10 mHz for FAM-BM (3.32 Ohm) compared to FAM (2.86 Ohm). A higher real part of impedance indicates higher resistance.

The lower imaginary part of impedance for FAM compared to FAM-BM at a frequency of 10 mHz is due to higher capacitance (Figure 3D). Indeed, the calculations of components of complex capacitances from the impedance data showed a higher real part of capacitance for FAM at low frequencies (Figure 3E). The frequency dependences of components of complex capacitance revealed relaxation-type dispersion [3,27], which was represented by a decrease in the real components (Figure 3E) with increasing frequency and maxima in the frequency dependences of the imaginary components (Figure 3F) at the relaxation frequencies. GCD data showed linear charge-discharge curves with longer charge-discharge times for FAM compared to FAM-BM at the same current densities (Figure 3G,H). As a result, the FAM electrodes showed higher capacitance compared to the capacitance of FAM-BM. The capacitance decreased with increasing current density (Figure 3I). The FAM and FAM-BM electrodes showed capacitances of 4.22 and 1.02 F cm^−2^, respectively, at a current density of 3 mA cm^−2^. The lower capacitance of electrodes prepared by high-energy ball milling is in contrast with the results of previous investigations [28] of γ-Fe_2_O_3_, which showed significantly higher capacitance of high-energy ball-milled material compared to original γ-Fe_2_O_3_ material. The lower capacitance and higher resistance of FAM-BM can result from the crystallization of FeOOH and its partial conversion to the Fe_2_O_3_ phase (Figure 2).

**Figure 4:**
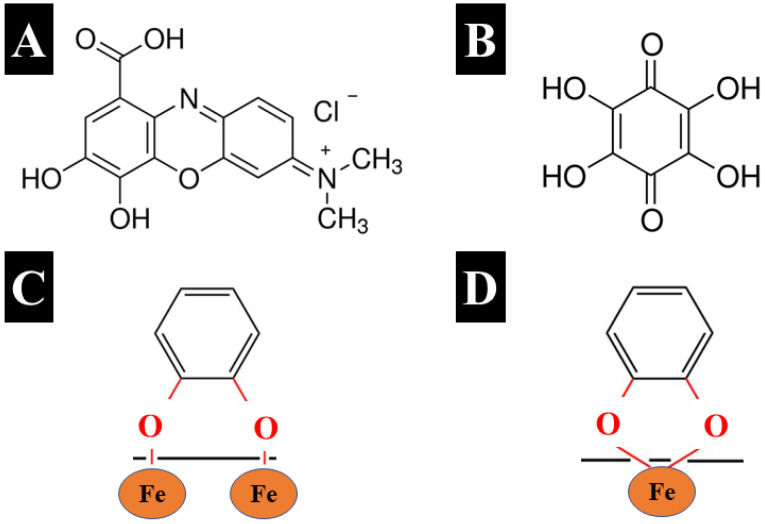
Chemical structures of (**A**) GC and (**B**) THB, and catecholate bonding mechanisms to Fe atoms on the particle surface: (**C**) bidentate bridging bonding and (**D**) bidentate chelating bonding.

Another strategy developed in this investigation was based on the use of capping agents for synthesis. Figure 4A,B shows the chemical structures of organic molecules used as capping agents for synthesis. GC and THB are aromatic molecules that belong to a catechol family of materials. Different molecules from the catechol family exhibit strong adhesion to inorganic surfaces by bidentate bridging (Figure 4C) or chelating mechanism (Figure 4D), similar to that of mussel proteins [29], which facilitate bonding to different surfaces. Strong catecholate-type bonding is critically important for THB and GC application as capping agents for synthesis. Capping agent adsorption can reduce particle growth.

The electric charge of the GC molecule facilitates electrostatic repulsion of particles containing adsorbed GC. The THB structure has multiple bonding sites, which can potentially be involved in catecholate-type bonding, including adjacent phenolic OH groups or adjacent phenolic OH groups and carbonyl groups. THB belongs to the quinone family of molecules. In contrast to other quinone molecules, which contain two redox-active groups and form a redox couple, the chemical structure of THB contains six redox-active groups [30]. The oxidation of THB results in transformation to rhodizonic acid and triquinoyl, and the reduction reaction involves transformation to hexahydroxybenzene [30]. The redox reactions involve three different couples of materials and redox transformations for each redox couple of compounds involve two redox-active groups [30].

Another approach was based on the use of PEI as an alkalizer and dispersing agent. Our investigation is motivated by the interest in avoiding the use of inorganic alkalis by the application of a more environmentally friendly alkalizer, which offers additional benefits of enhanced nanoparticle dispersion. PEI is a biocompatible polymer that exhibits a strong “proton sponge” effect and forms aqueous solutions with high pH [31]. The multiple amino groups of PEI adsorb protons, resulting in the large buffering capacity of this polymer [31]. PEI is widely used as a dispersant for inorganic materials of different types [32,33,34,35] and carbon nanotubes [36]. Moreover, PEI is a promising co-dispersant for materials of different types.

**Figure 5:**
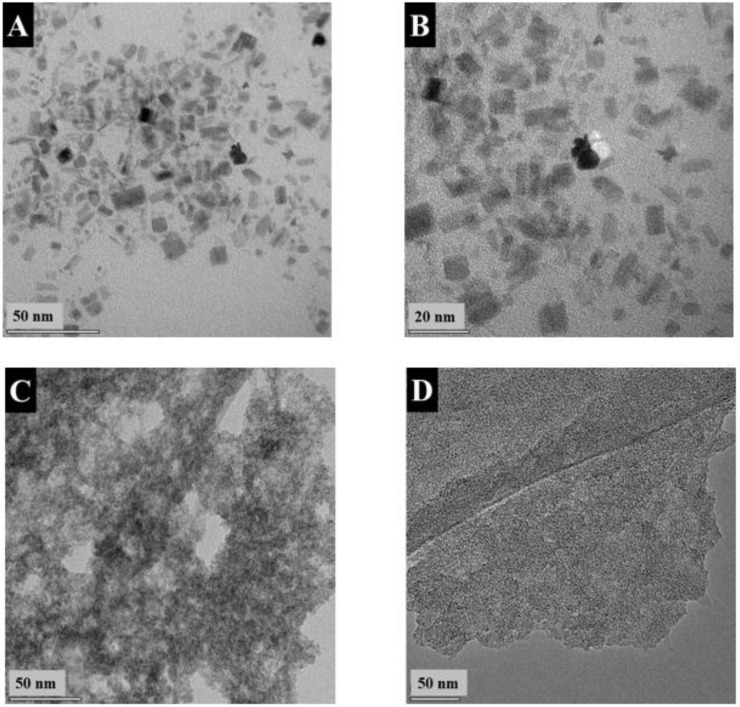
TEM images of (**A**,**B**) FAM-THB at different magnifications: (**A**) ×310,000 and (**B**) 510,000; (**C**) FAM-GC at a magnification of ×240,000 and (**D**) FAM-PEI at a magnification of ×75,000.

The XRD pattern of FAM-THB showed small peaks of α -FeOOH. However, the material contained a large amount of amorphous phase (Figure 2c). The FAM-GC and FAM-PEI were amorphous (Figure 2d,e). The TEM images at different magnifications (Figure 5A,B) showed that FAM-THB contained small non-agglomerated particles with a typical size below 20 nm (Figure 5B). The formation of large particles, such as particles shown in Figure 1A,B, was avoided. The TEM images show the benefits of dispersants for the fabrication of nanoparticles. It is suggested that dispersant adsorption on FeOOH during synthesis limited particle growth.

The TEM images of FAM-THB, FAM-GC and FAM-PEI showed that the formation of large particles, similar to those shown in Figure 1, was avoided. This resulted in improved suspension stabilities. However, the drying of the FAM-GC and FAM-PEI particles resulted in their agglomeration. The size of the primary FAM-GC and FAM-PEI particles was about 10 and 50 nm, respectively. It is suggested that multiple amino groups of PEI promote particle bridging.

**Figure 6:**
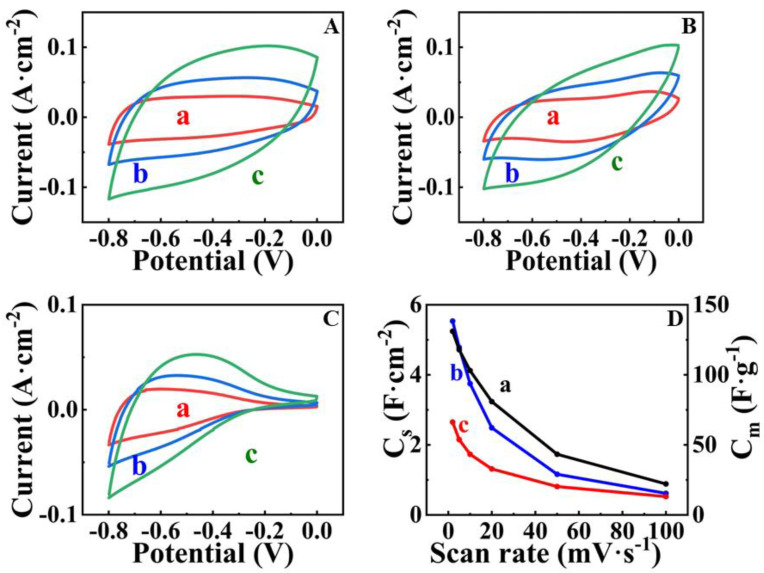
(**A**–**C**) CVs at scan rates of (a) 5, (b) 10 and (c) 20 mV s^−1^ for (**A**) FAM-THB, (**B**) FAM-GC and (**C**) FAM-PEI; (**D**) capacitance versus scan rate for (a) AM-THB, (b) FAM-GC and (c) FAM-PEI. SCE was used as a reference electrode.

**Figure 7:**
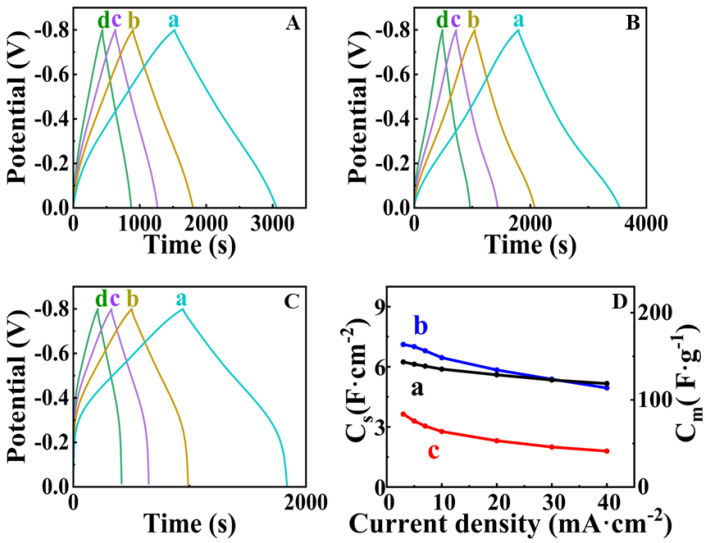
(**A**–**C**) GCD data at current densities of (a) 3, (b) 5 and (c) 7 and (d) 10 mA cm^−2^ for (**A**) FAM-THB, (**B**) FAM-GC and (**C**) FAM-PEI; (**D**) capacitance versus current density for (a) AM-THB, (b) FAM-GC and (c) FAM-PEI. SCE was used as a reference electrode.

Figure 6 shows cyclic voltammetry data for FAM-THB, FAM-GC and FAM-PEI. The CVs for FAM-GC and FAM-THB exhibit improved shape compared to FAM and FAM-BM as a result of enhanced charge-discharge currents in the range of −0.2–0 V. The CVs obtained at lower scan rates did not show redox peaks, which can result from redox reactions of THB or GC. It should be noted that the amounts of THB and GC were very low. THB and GC were used as capping agents, and we did not detect their contribution to the electrode capacitance. However, the shape of the CVs for FAM-PEI shows low currents in the range of −0.2–0 V. Such a shape can be attributed to high charge transfer resistance in this potential range [17]. The problem can be addressed by the reduction of particle size or by the formation of composites [7,17]. The capacitances for FAM-THB, FAM-GC and FAM-PEI were found to be 5.24, 5.53 and 2.65 F cm^−2^ at a scan rate of 2 mV s^−1^. However, FAM-THB showed better capacitance retention with increasing scan rate and highest capacitance at scan rates above 20 mV s^−1^ compared to FAM-GC and FAM-PEI. Figure 7 shows GCD data for FAM-THB, FAM-GC and FAM-PEI. The charge-discharge curves for FAM-THB and FAM-GC were nearly linear, whereas the charge-discharge curves for FAM-PEI deviated from the linear shape. The capacitances calculated from the GCD data at a current density of 3 mA cm^−2^ for FAM-THB, FAM-GC and FAM-PEI were 5.74, 6.54 and 3.35 F cm^−2^, respectively.

**Figure 8:**
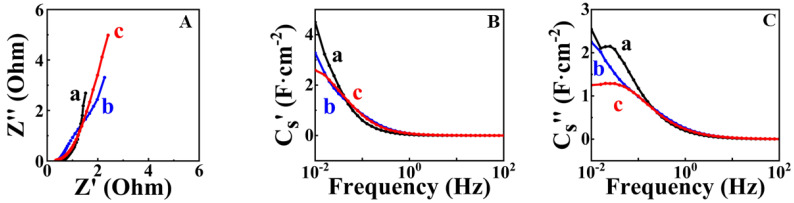
(**A**) Nyquist plot of EIS data; (**B**) real C_S_′ and (**C**) imaginary C_S_″ components of complex capacitance for (a) FAM-THB, (b) FAM-GC and (c) FAM-PEI.

FAM-THB showed better capacitance retention compared to FAM-GC and FAM-PEI. Figure 8 shows EIS data for FAM-THB, FAM-GC and FAM-PEI.

The electrodes showed lower resistance compared to FAM and FAM-BM electrodes. The lowest resistance and highest capacitance at low frequencies were obtained for FAM-THB. Table 1 compares the literature data on capacitances of high active mass FeOOH electrodes in Na_2_SO_4_ electrolyte with the results of this investigation. It should be noted that high active mass loading is critical for practical applications. It is known [20] that for activated carbon electrodes, the mass loading must be at least 10 mg cm^−2^, whereas for inorganic materials with higher density, the mass loading must be at least 40 mg cm^−2^. It is challenging to achieve high active mass loading and good electrode performance at high active mass loading. The nanotechnologies developed in this investigation facilitated the fabrication of electrodes with high active mass loading and high areal capacitance. Many investigations have focused on the fabrication of electrodes with low active mass [20,37]. In Table 1, we summarized data for electrodes with an active mass of 40 mg cm^−2^ or close to the value of this active mass loading.

The capacitance obtained for FAM-PEI is comparable with the capacitances obtained in previous investigations [17,38,39], where inorganic alkalis were used for wet chemical synthesis. The results of this investigation show the feasibility of a conceptually new approach for the synthesis of nanoparticles from aqueous solutions of metal salts using an organic alkalizer. However, the capacitance of FAM-PEI is lower than that obtained in a combined chemical precipitation and liquid-liquid extraction method, which was based on the use of 16-phosphonohexadecanoic acid as a capping agent for synthesis and an extractor for liquid-liquid extraction [7]. The ability to avoid the use of an inorganic alkali offers environmental benefits. Moreover, the reduction of ionic strength of the solution for synthesis by replacing inorganic alkali with PEI offers benefits for the improved colloidal stability of the prepared nanoparticles. It is suggested that PEI acted as a dispersant and prevented the formation of large particles (Figure 1). Therefore, this approach is promising and further improvements can be achieved by combining this method with liquid-liquid extraction or capping agents. The capacitance achieved using THB as a capping agent was comparable to that reported for the combined method [7]. However, the approach based on the use of THB offers the benefits of a simple fabrication procedure. The capacitance obtained using GC was higher than that obtained by the combined method [7], based on the use of 16-phosphonohexadecanoic acid as a capping agent for synthesis and an extractor for liquid-liquid extraction. This finding indicates that GC is an efficient capping agent for synthesis, which allows for higher capacitance using a simple electrode preparation procedure. The catechol ligand of this molecule and electric charge were critically important for the synthesis of non-agglomerated FeOOH nanoparticles, their dispersion and efficient mixing with MWCNT for the fabrication of nanocomposite anodes. The comparison with the literature [12,13] indicates that FAM-THB and FAM-GC electrodes showed improved pseudocapacitive CV and GCD shapes and reduced impedance. It should be noted that the capacitances of anodes reported in the literature are typically lower than the capacitances of cathodes, such as MnO_2_. This results in a lower total capacitance of asymmetric devices. The capacitance achieved in this investigation for FeOOH cathodes is comparable with the capacitance of MnO_2_ anodes of a similar mass [26].

## 4. Conclusions

The FeOOH powders prepared by the chemical precipitation method using inorganic alkali contained large particles with a typical size of about 0.5–2 μm, which are detrimental for FeOOH applications in anodes of supercapacitors. Various strategies have been used for the prevention of large particle formation. HEBM resulted in enhanced FeOOH crystallization and partial transformation to Fe_2_O_3_. As a result of crystallization and phase transformation, the obtained material showed reduced capacitance. The use of THB and GC as capping agents or PEI as alkalizers-dispersants eliminated the formation of large particles and facilitated the formation of nanostructured FeOOH. The highest capacitances obtained using FAM-THB, FAM-GC and FAM-PEI were 5.74, 6.54 and 3.35 F cm^−2^, respectively, at a current density of 3 mA cm^−2^. This investigation demonstrated the feasibility of preparing FeOOH using a conceptually new approach, which is based on the use of PEI as an alkalizer and dispersant. This approach is promising and can be combined with other strategies that are based on capping agents or liquid-liquid extraction. The anodes prepared in this investigation are promising for the fabrication of asymmetric devices because the capacitance obtained is comparable with the capacitance of advanced cathodes, such as MnO_2_. Therefore, the problem of lower capacitance of anode materials compared to cathode materials can be avoided.

## Figures and Tables

**Table 1:** Real capacitance of high active mass loading FeOOH electrodes in 0.5 M Na_2_SO_4_ electrolyte.

Potential Range, V vs. SCE	Mass Loading, mg cm^−2^	C_S_, F cm^−2^	Reference
−0.8–0	36	2.4	[38]
−0.8–0	39.6	3.3	[39]
−0.8–+0.1	36	3.5	[17]
−0.8–0	37	5.86	[7]
−0.8–0	40	4.5	[11]
−0.8–0	40 (FAM-PEI)	3.35	This work
−0.8–0	40 (FAM-THB)	5.74	This work
−0.8–0	40 (FAM-GC)	6.54	This work

## Data Availability

Data contained within the article are available.
